# Association rare: Syndrome de Sjögren et liposarcome de la parotide

**DOI:** 10.11604/pamj.2015.20.420.6212

**Published:** 2015-04-29

**Authors:** Mahfoudhi Madiha, Khamassi Khaled

**Affiliations:** 1 Service de Médecine Interne A, Hôpital Charles Nicolle, Tunis, Tunisie; 2 Service ORL, Hôpital Charles Nicolle, Tunis, Tunisie

**Keywords:** syndrome de Sjögren, liposarcome, parotide, Sjogren Syndrome, liposarcoma, parotide

## Image en medicine

Les liposarcomes de la parotide ont été rarement rapportés chez l'adulte. Aucune association à un syndrome de Sjögren n'a été décrite dans la littérature. Patiente âgée de 48 ans, suivie pour un syndrome de Sjögren depuis 5 ans, était hospitalisée pour une tuméfaction cervicale gauche évoluant depuis 3 mois. Le syndrome de Sjögren a été retenu devant l'association d'une xérostomie, une xérophtalmie, une kératoconjonctivite au test de Rose Bengal, des anti-SSA positifs et une sialadénite stade IV de chisholm. Il était primitif vue l'absence d'une connectivite ou d'une autre pathologie associée. A l'examen, elle avait une tuméfaction de la région parotidienne gauche de 4 cm de diamètre, ferme, indolore, peu mobile, sans signes inflammatoires en regard. Le reste de l'examen ORL était sans anomalies. Elle n'avait pas d'adénopathies périphériques. L'examen biologique a retrouvé une hypergammaglobulinémie polyclonale. Une poussée de sa maladie, un lymphome ou une cause infectieuse étaient évoqués. L'échographie cervicale a révélé une formation parotidiennee hypoéchogène hétérogène bien limitée au niveau de la loge parotidienne inférieure gauche. L'IRM parotidienne a objectivé un processus hétérogène en hyposignal T1, hypersignal T2, se réhaussant après injection de gadolinium. Le bilan d'extension était négatif. Le Traitement a consisté en une parotidectomie totale gauche associée à un curage ganglionnaire homolatéral et une radiothérapie post-opératoire. L'examen extemporané était évocateur d'un liposarcome. L'examen anatomopathologique a conclut à un liposarcome dédifférencié myxoïde de haut grade de malignité. L'évolution était marquée par une rémission totale pour un recul de 3 ans.

**Figure 1:**
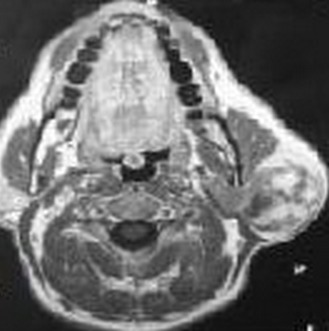
IRM parotidienne (coupe axiale, T1): processus hétérogène parotidien gauche en hyposignal T1

